# Toll-like receptor 2 polymorphism in juvenile dermatomyositis

**DOI:** 10.1186/1546-0096-9-S1-O40

**Published:** 2011-09-14

**Authors:** P Mathiesen, C Enevold, T Lange, B Kornblit, S Nielsen, K Müller

**Affiliations:** 1 Dept.of Paediatrics, Holbaek University Hospital, Denmark; 2 Institute for Inflammation Research, Dept. of Rheumatology, Rigshospitalet, University Hospital of Copenhagen, Denmark; 3 Dept. of Biostatistics, University of Copenhagen, Denmark; 4 Laboratory of Molecular Medicine, Dept. of Clinical Immunology, Rigshospitalet and Allogeneic Hematopoietic Cell Transplantation Laboratory, University Hospital of Copenhagen, Denmark; 5 Dept. of Hematology, Rigshospitalet,Paediatric Clinic II, Rigshospitalet, University Hospital of Copenhagen, Denmark

## Background

Juvenile dermatomyositis (JDM) is characterised by chronic inflammation in skeletal muscle, skin, and other target organs. Prognosis has improved in the past 10-20 years, but it is still a disease with high morbidity and considerable sequelae such as muscle weakness, joint contractures, calcinosis and lipodystrophy. There is increasing evidence that single nucleotide polymorphisms (SNPs) are associated with chronic inflammatory diseases. In the present study, we investigated known SNPs within genes that previously have been associated with inflammatory myopathies, including JDM and dermatomyositis.

## Aim

The aim of the study was to investigate known polymorphisms within the genes encoding Toll-like receptor (TLR)1-10; nucleotide binding oligomerisation domain (NOD)1-2; interferon-induced helicase C domain-containing protein 1 (IFIH1); and melanoma differentiation associated gene 5 (MDA5), as well as single nucleotide polymorphisms (SNPs) in tumour necrosis factor (TNF)-alpha and protein tyrosine phosphatase, non-receptor type 22 (PTPN22) and their association with the disease course of Juvenile Dermatomyositis (JDM) in a population-based follow-up study.

## Methods

In 53 JDM patients SNPs in the pattern recognition receptor genes encoding TLR1-10, DDX58 (RIG-I), IFIH1 (MDA5), NOD1 (CARD4), and NOD2 (CARD15) were analysed using an in-house multiplex bead-based assays. The TNF-alpha (rs1800629) and PTPN22 (rs2476601) polymorphisms were genotyped by pyrosequencing.

## Results

The TLR2-rs1898830 SNP was found to be associated with calcinosis (p=0.006) and lipodystrophy (p=0.0006). Patients with the G/G genotype had a significantly higher risk (OR: 60.0, CI: 5.0-719.0) of developing lipodystrophy than patients with the A/A or A/G genotypes.

No other SNPs were significantly associated with JDM outcome.

## Conclusion

The present study suggests an association between TLR2-rs1898830 and disease outcome in JDM.

